# Development of an HPLC-UV Method for the Analysis of Drugs Used for Combined Hypertension Therapy in Pharmaceutical Preparations and Human Plasma

**DOI:** 10.1155/2013/179627

**Published:** 2013-03-24

**Authors:** Serife Evrim Kepekci Tekkeli

**Affiliations:** Department of Analytical Chemistry, Faculty of Pharmacy, Bezmialem Vakif University, Fatih, 34093 Istanbul, Turkey

## Abstract

A simple, rapid, and selective HPLC-UV method was developed for the determination of antihypertensive drug substances: amlodipine besilat (AML), olmesartan medoxomil (OLM), valsartan (VAL), and hydrochlorothiazide (HCT) in pharmaceuticals and plasma. These substances are mostly used as combinations. The combinations are found in various forms, especially in current pharmaceuticals as threesome components: OLM, AML, and HCT (*combination* I) and AML, VAL, and HCT (*combination* II). The separation was achieved by using an RP-CN column, and acetonitrile-methanol-10 mmol orthophosphoric acid pH 2.5 (7 : 13 : 80, v/v/v) was used as a mobile phase; the detector wavelength was set at 235 nm. The linear ranges were found as 0.1–18.5 **μ**g/mL, 0.4–25.6 **μ**g/mL, 0.3–15.5 **μ**g/mL, and 0.3–22 **μ**g/mL for AML, OLM, VAL, and HCT, respectively. In order to check the selectivity of the method for pharmaceutical preparations, forced degradation studies were carried out. According to the validation studies, the developed method was found to be reproducible and accurate as shown by RSD ≤6.1%, 5.7%, 6.9%, and 4.6% and relative mean error (RME) ≤10.6%, 5.8%, 6.5%, and 6.8% for AML, OLM, VAL, and HCT, respectively. Consequently, the method was applied to the analysis of tablets and plasma of the patients using drugs including those substances.

## 1. Introduction

**Figure 1:**
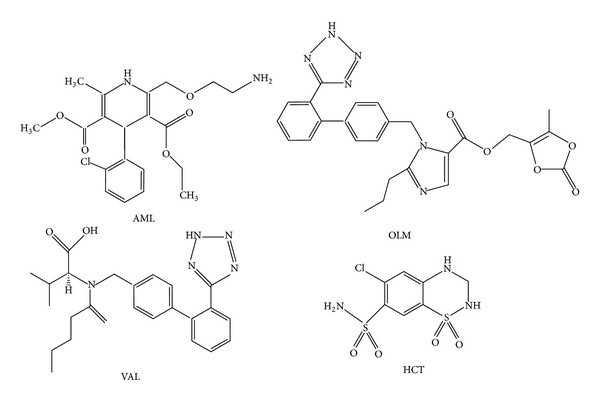
Chemical structures of analyzed drug substances.

Hypertension currently affects more than 1 billion adults worldwide, and by 2025, the projected estimate is 1.5 billion [1]. Calcium channel blockers (CCBs), angiotensin receptor blockers (ARBs), angiotensin converting enzyme (ACE) inhibitors, and diuretics are generally used for hypertension therapy [2–4]. Amlodipine besilate (AML), chemically, 3-ethyl-5-methyl(4RS)-2-[(2-aminoethoxy)methyl]-4-(2-chlorophenyl)-methyl-1-dihydropyridine-3,5-dicarboxylate benzenesulfonate, is a long acting CCB [5]. Olmesartan medoxomil (OLM), chemically, 5-methyl-2-oxo-2H-1,3-dioxol-4-yl)methyl 4-(2-hydroxypropan-2-yl)-2-propyl-1-({4-[2-(2H-1,2,3,4-tetrazol-5-yl)phenyl]phenyl}methyl)-1H-imidazole-5-carboxylate and valsartan (VAL) 2S-3-methyl-2-[N-({4-[2-(2H-1,2,3,4-tetrazol-5-yl)phenyl]phenyl}methyl) pentanamido] butanoic acid are the ARBs also known as angiotensin receptor II antagonists (ARA-IIs) [6, 7]. Hydrochlorothiazide (HCT), 6-chloro-1,1-dioxo-3,4-dihydro-2*H*-1,2,4-benzothiadiazine-7-sulfonamide, is a diuretic substance which is often used in combination with other antihypertensive drugs such as CCBs, ACE inhibitors, or more recently ARBs [8–11]. Chemical structures were given in Figure 1.

The effective treatment of moderate or severe hypertension often requires the use of multiple antihypertensive agents from different drug classes [12–16]. AML, OLM, VAL, and HCT are used as combinations in pharmaceutical preparations, for the treatment of hypertension and cardiovascular diseases [17, 18]. The literature survey revealed that a number of methods have been reported for the determination of AML, OLM, VAL, and HCT individually or in combination with each other or other drug substances [19–36]. HPLC has been the major technique used for these assays. In the literature, there is no method that enables the simultaneous determination of the current drug formulations of the substances: OLM, AML, and HCT (*combination* I) and AML, VAL, and HCT (*combination* II). This paper describes a rapid and stability-indicating HPLC-UV method for the determination of *combination* I and *combination* II in pharmaceutical formulations and plasma samples. For plasma samples, before the chromatographic process, a liquid-liquid extraction (LLE) procedure was carried out, and high recovery values were achieved. The proposed HPLC method was successfully applied to plasma samples obtained from 8 hypertensive patients after oral administration of these antihypertensive drug substances. 

## 2. Experimental

### 2.1. Apparatus

The HPLC analyses were performed on a Thermo Separation Products Liquid Chromatograph (TX, USA) which consisted of a P4000 solvent delivery system equipped with a Rheodyne injection valve with a 20 *μ*L loop, a UV3000 detector set at 235 nm, and an SN4000 automation system software. Chromatographic separation was achieved isocratically at 30°C on an ACE (Advanced Chromatography Technologies, UK) CN (cyano) column (cyano groups bounded to silica surface of the column) with the dimensions 4.6 mm I.D, 200 mm length, and 5 *μ*m particle size. The mobile phase was an acetonitrile-methanol-10 mM phosphoric acid (pH 2.5) (7 : 13 : 80, v/v/v) with a flow rate of 1.0 mL/min. 

### 2.2. Reagents and Solutions

AML, OLM, VAL, HCT, donepezil HCl, acetylcystein, metformin HCl, atorvastatin, naproxen sodium, and cilostazol were kindly supplied by Abdi Ibrahim Ilaç (Istanbul, Turkey). Tizanidine and telmisartan were obtained from Novartis. The pharmaceutical preparations of the investigated substances Sevikar HCT (40 mg olmesartan medoxomil, 10 mg amlodipine besilat, and 25 mg hydrochlorothiazide) and Exforge HCT (10 mg amlodipine besilat, 320 mg valsartan, and 25 mg hydrochlorothiazide) were obtained from a drug store. All chemicals and reagents were of an analytical grade. 

Stock solutions of the drug substances (1 mg/mL) were prepared in methanol. Prior to measurements, stock solutions of AML, OLM, VAL, and HCT were diluted with acetonitrile-methanol-water (7 : 13 : 80, v/v/v) so as to prepare the working standard solutions of 100 *μ*g/mL and 1 *μ*g/mL. Various dilutions were made to prepare working solutions. HPLC analysis was carried out with 20 *μ*L aliquots of various concentrations of the working solutions. 

### 2.3. Assay Procedure for Pharmaceutical Preparations

Five tablets of each preparation (Sevikar HCT which includes *combination *I and Exforge HCT which includes *combination* II) were weighed and finely powdered. The powder equivalent to 4 mg OLM, 1 mg AML, and 2.5 mg HCT for *combination* I and 1 mg AML, 32 mg OLM, and 2.5 mg HCT for *combination* II was accurately weighed and transferred to 100 mL volumetric flasks. 75 mL of methanol was transferred to each volumetric flask, and then extractions were performed mechanically for 20 minutes and sonicated for 20 more minutes. The dilutions were made with methanol to give a solution containing 40 *μ*g/mL OLM, 10 *μ*g/mL AML, and 25 *μ*g/mL HCT (for *combination* I) and 10 *μ*g/mL AML, 320 *μ*g/mL VAL, and 25 *μ*g/mL HCT (for *combination* II). From each of these solutions, 1.0 mL of the extract was transferred to a 10 mL volumetric flask. The extracts were diluted with acetonitrile-methanol-water (7 : 13 : 80, v/v/v/) to the mark to give the working tablet solutions containing 4 *μ*g/mL OLM, 1 *μ*g/mL AML, and 2.5 *μ*g/mL HCT (for *combination* I) and 1 *μ*g/mL AML, 32 *μ*g/mL VAL, and 2.5 *μ*g/mL HCT (for *combination* II). 20 *μ*L of the sample from each working tablet solution was directly injected into the HPLC column. All measurements were repeated six times for each concentration. The nominal contents of pharmaceutical preparations were calculated using the regression equation of the calibration graph. The related calibration curve was prepared by the analysis of the working solutions of the drug substances. The calibration curve equation is *y* = *ax* + *b*, where *y* represents the peak areas and *x* represents the concentrations of the drug substances.

### 2.4. Selectivity of the Method for Tablet Analysis

In order to develop a stability-indicating method, forced degradation (stress testing) is undertaken to demonstrate selectivity, particularly when little information is available about potential degradation products [37]. 

The selectivity of the proposed method for tablet analyses was determined by checking the peak purities of the related drug substances during the force degradation studies. 

The stress conditions were as follows.

*Hydrolysis.* Individually, 5 mg of the drug substances was dissolved in 5 mL of methanol in a 10 mL volumetric flask and heated for 1 h at 80°C after adding: (a) 5 mL of water for neutral hydrolysis, (b) 5 mL of 1 N HCl for acid hydrolysis, and (c) 5 mL of 1 N NaOH for basic hydrolysis.

*Chemical Oxidation. *5 mg of the drug substances was dissolved in 5 mL of methanol in a 10 mL volumetric flask, and 100 *μ*L of 30%  H_2_O_2_ solution (v/v) was added and mixed. The solution was left at room temperature for 1 hour in the dark.

*Photochemical Degradation. *5 mg of the drug substances was dissolved in 5 mL of methanol in a 10 mL volumetric flask, and the solution was exposed to direct sunlight for 8 hr at 20°C.

Each of the stressed solutions was diluted with the acetonitrile-methanol-water (7 : 13 : 80, v/v/v) to obtain a theoretical concentration of 1 *μ*g/mL. Each solution was analyzed three times. 

### 2.5. Assay Procedure for Plasma Samples

Plasma sample collection and preparation: plasma samples were collected from 8 hypertensive patients after oral administration of the investigated antihipertansive drug substances. The main characteristics of the patients and the drugs they used are summarized in Table 1.

Drug-free human plasma samples were obtained from the Blood Bank of Bezmialem Vakif University (Istanbul, TURKEY) and stored in polypropylene tubes at −20°C until analysis. Blood samples were collected into the tubes containing disodium EDTA (ethylenediaminetetraacetic acid) and centrifuged at 4500 rpm for 10 min. 1 mL of the resultant plasma was spiked with various concentrations of working solutions of the drug substances. Each plasma sample was basified with 0.5 mL of aqueous 0.1 M NaOH solution. Then, the analytes were extracted from plasma using 5 mL of n-hexane-ethylacetate-isoamyl alcohol (88 : 10 : 2, v/v/v) and vortex mixing for 2 min. The samples were centrifuged for 1 min at 1500 rpm. For each sample, the organic phase was transferred into another tube for evaporation at 45°C, under nitrogen, and the residue was dissolved in 0.5 mL of mobile phase solution. The samples were filtered through a 0.22 membrane filter before injection into the HPLC column. Plasma samples were quantified using the peak area of the analytes. Each plasma sample was analyzed for three times.

### 2.6. Method Validation

The validation of the method was carried out according to the guidelines given by the FDA [38] and the ICH [39]. In this way, recovery, linearity, working range, intra- and interday accuracy and precision, LOQ (limit of quantitation), LOD (limit of detection), selectivity, and stability studies were tested for each analyte. 

### 2.7. Calibration Curves for Plasma Analysis, LOD, and LOQ

Calibration curves were prepared by the analysis of 1 mL of human blank plasma samples spiked with various concentrations of working solutions of the drug substances. The samples were then submitted to the processes such as extraction, chromatographic separation, and UV detection described above. Calibration curves were obtained by linear least-squares regression analysis plotting of peak areas versus the concentrations. The calibration curve equation is *y* = *ax* + *b*, where *y* represents the peak areas and *x* represents the concentrations of the drug substances.

LOD was determined as the lowest concentration giving a signal to noise ratio (S/N) of 3 for all of the drug substances. LOQ, the lowest amount of analyte that can be quantified with acceptable precision and accuracy, was determined as S/N of 10. 

### 2.8. Precision and Accuracy

Precision and accuracy of the method for intraday and interday plasma analyses were determined by studying with the QC (quality control) samples at three different concentration levels (low, medium, and high) for each drug. For intra-day investigation, six replicates of samples for each drug at each QC level were analyzed in the same day. Interday precision and accuracy values were determined by studying the samples every day during five consecutive days. Six replicates at each concentration were assayed per day.

### 2.9. Recovery and Stability

Absolute recoveries of the drugs at three QC levels were measured by comparing the peak areas of each drug obtained from the plasma with peak areas obtained by the direct injection of pure aqueous drug standards. The relative recoveries of the drugs at three QC levels were calculated by comparing the found concentrations obtained from the drugs spiked with plasma to the actually added concentrations.

The stability of the working solution (in acetonitrile-methanol-water (7 : 13 : 80, v/v/v)) of each drug substance was tested at several storage conditions (at room temperature for 2 weeks and 4°C for 1 month). The stabilities of the drug substances in the extraction solvent were also investigated (at room temperature for 1 day and 4°C for 1 week). The freeze-thaw stability of the drug substances in plasma samples was evaluated over five freeze-thaw cycles. Plasma samples in three QC levels were immediately frozen at −20°C and thawed at room temperature for five consecutive times. After that, the samples were processed and assayed. In order to determine the stability of the drug substances in plasma, the spiked plasma samples were stored at room temperature for 24 h and −20°C for 2 weeks, and the evaluations were carried out at intervals. Long-term stability was assessed using the samples stored at −20°C over a period of 8 weeks. 

### 2.10. Selectivity of the Method for Plasma Analysis

Selectivity of the method was tested by analyzing blank human plasma samples from 8 different sources and by comparing them with the spiked plasma samples under optimized chromatographic conditions.

## 3. Results and Discussion

### 3.1. Optimization of Chromatographic Conditions

**Figure 2:**
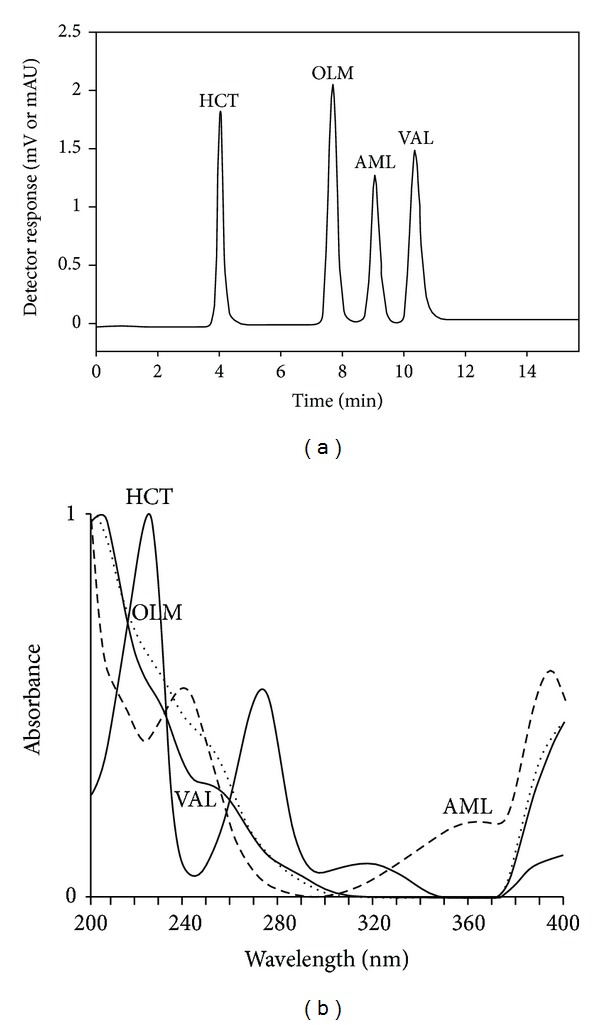
(a) The chromatogram of 1 *μ*g/mL OLM, VAL, AML, and HCT. (b) UV spectrum of 1 *μ*g/mL OLM, VAL, AML, and HCT in mobile phase.

Reversed-phase HPLC-UV method was preferred for the determination of AML, OLM, VAL, and HCT. Preliminary experiments were carried out to achieve the best chromatographic conditions for the simultaneous determination of the drug substances. Several column types and lengths were trialed considering other chromatographic parameters. 25 cm CN column with a 4.6 mm inner diameter and a 5 *μ*m particle size was chosen. Acetonitrile, methanol, and phosphoric acid were used as basic constituents of examined mobile phases. Different proportions of these solvents were tested. The best separation was achieved by the isocratic elution system using acetonitrile-methanol-10 mM phosphoric acid (pH 2.5) (7 : 13 : 80, v/v/v) with a flow rate of 1.0 mL/min. A UV detector was set at 235 nm (Figure 2(a)). Under these conditions, elution of analytes was completed in less than 12 min. Retention times were as follows: for HCT *t*_*r*_ = 4.00 ± 0.15 min, OLM *t*_*r*_ = 7.09 ± 0.11 min, AML *t*_*r*_ = 9.02 ± 0.13 min, and VAL *t*_*r*_ = 10.02 ± 0.09 min (Figure 2(b)). The chromatograms were evaluated on the basis of peak areas of the drug substances. 

## 4. Application to Pharmaceutical Preparations

### 4.1. Calibration Curve

The regression equations were *y* = 27712*x* + 753.5 (*r* = 0.9996) for AML, *y* = 27450*x* + 677.3 (*r* = 0.9997) for OLM, *y* = 28691*x* + 503.8 (*r* = 0.9999) for VAL, and *y* = 20450*x* + 862.6 (*r* = 0.9998) for HCT, respectively. For all the compounds, the coefficients of determination (*r* values) prove that the method was linear in the specified range.

### 4.2. Recovery and RSD% of Tablet Extraction

The recovery values of the extraction procedure with methanol were 97.3%, 93.5%, 95.8%, and 98.9%, the RSD% values were 1.35, 2.42, 1.67, 3.72 for AML, OLM, VAL, and HCT (*n* = 6).

### 4.3. Selectivity of the Method for Tablet Analysis

**Figure 3:**
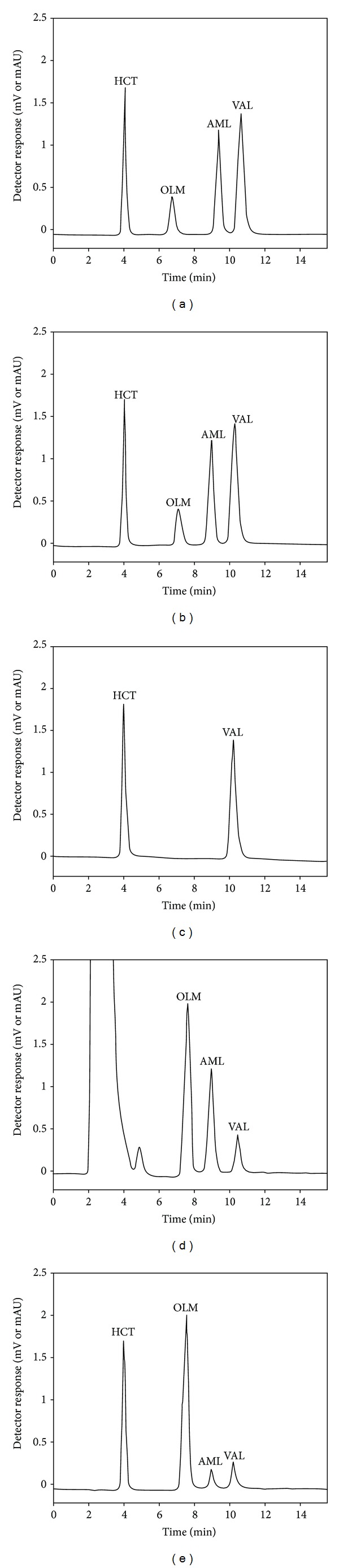
Chromatograms corresponding to drug solutions subjected to (a) neutral hydrolysis, (b) acid hydrolysis, (c) alkaline hydrolysis, (d) chemical oxidation, and (e) exposure to sunlight.

For tablet analyses, selectivity was assessed immediately after AML, OLM, VAL, and HCT solutions were exposed to neutral, acidic, and basic hydrolysis and chemical oxidation with H_2_O_2_ and sunlight. As seen in the chromatograms in Figure 3, by the reason of neutral and acidic hydrolysis, OLM decomposed about 71%. Additionally, because of alkali hydrolysis the peaks of AML and OLM disappeared, indicating that the substances totally decomposed. Chemical oxidation caused about 61% decomposition of VAL, and a huge meaningless peak occurred instead of HCT showing a complete decomposition, and at about 5 min, a degradation product with a little peak appeared. 64% of AML and 60% of VAL decomposed due to the exposition to sunlight. The peak purities of the parent drugs were also confirmed by their UV spectra. 

### 4.4. Plasma Analyses

#### 4.4.1. Sample Cleanup for Plasma Analyses

In the initial studies, for protein precipitation some trials were conducted with acetonitrile, methanol, and perchloric acid; however, ion suppression and lower recovery values (<60%) were observed. Therefore, the LLE procedure was preferred to remove the sample matrix and gain the investigated drug substances. The mixture of n-hexane-ethylacetate-isoamyl alcohol (88 : 10 : 2, v/v/v) was selected as an LLE solvent to extract the substances from the spiked plasma. 

#### 4.4.2. Assay Validation for Plasma Analysis

*Calibration Curves, LOD, and LOQ.* Linearity was established by least-squares linear regression analysis of the calibration curve. The constructed calibration curves were linear over the concentration range of 0.1–18.5 *μ*g/mL, 0.4–25.6 *μ*g/mL, 0.3–15.5 *μ*g/mL, and 0.3–18 *μ*g/mL for AML, OLM, VAL, and HCT, respectively. Peak areas of these drug substances were plotted versus their respective concentrations and linear regression analysis performed on the resultant curves. The regression equations were *y* = 34770*x* + 10953 (*r* = 0.9998) for AML, *y* = 31947*x* + 5036.2 (*r* = 0.9995) for OLM, *y* = 36544*x* + 1005.4 (*r* = 0.9998) for VAL, and *y* = 25436*x* + 5846.7 (*r* = 0.9999) for HCT. For all the compounds, the correlation coefficient values (*r* values) prove that the methods were linear in the specified range. All linearity parameters are summarized in Table 2.

The LOD values were found as 0.3, 0.08, 0.1, and 0.2 ng/mL, which is the concentration that yields a S/N of 3 : 1, and the LOQ values were 0.1, 0.4, 0.3, and 0.3, *μ*g/mL for AML, OLM, VAL, and HCT, respectively.

*Precision and Accuracy.* The QC samples at three concentration levels were analyzed with the method mentioned above. The results of precision and accuracy of the assay are summarized in Table 3. RSD values of both intraday and interday analyses were less than 4.2%, 3.5%, 5.8%, and 3.6%, and the relative mean error (RME) values were less than 7.4%, 3.3%, 3.9%, and 5.5%, for AML, OLM, VAL, and HCT, respectively. These results indicate that the method is reliable and reproducible.

*Recovery and Stability*. The absolute and relative recoveries were calculated for each analyte in low, medium and high concentrations (*n* = 6). Absolute recoveries were found between 75% and 80.3%, relative recoveries were found between 95.7% and 99.6% as shown in Table 4. 

The stability of the drug substances in human plasma and in acetonitrile-methanol-water (7 : 13 : 80, v/v/v) was investigated as described in Section 2. The analytes were found to be stable in human plasma for 30 days at −20°C and in acetonitrile-methanol-water (7 : 13 : 80, v/v/v) for 24 h at room temperature (<6% reduction) and for 1 week at 4°C. Besides, substances were stable in the extraction solvent for 8 hours at room temperature and for 48 hours at 4°C. The analytes were also found to be stable after three freeze-thaw cycles with a reduction of less than 5.66%.

**Figure 4:**
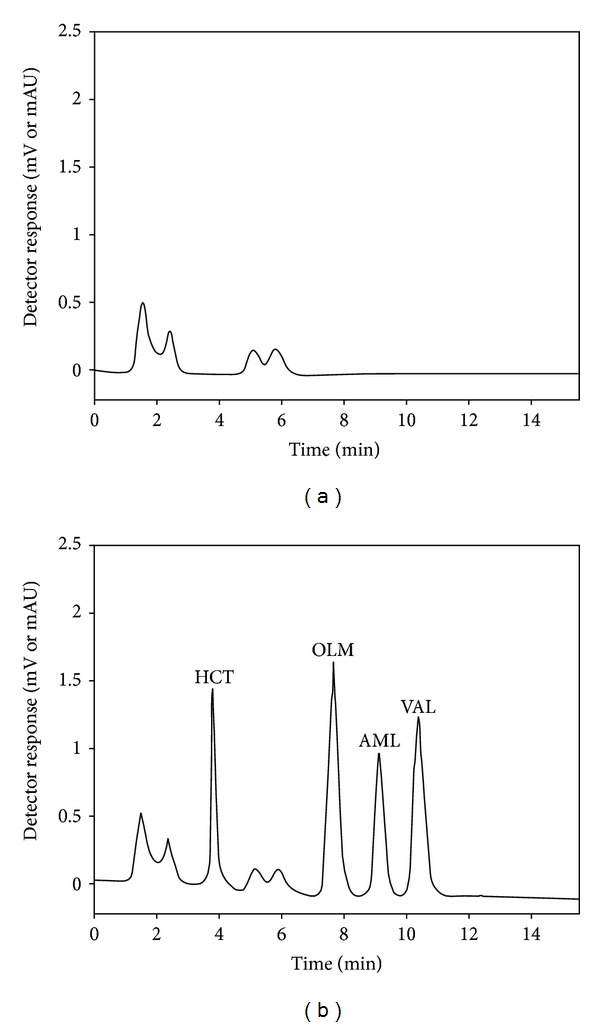
Chromatograms of (a) blank plasma and (b) a plasma sample of 1 mL spiked with 1 *μ*g/mL of OLM, VAL, AML, and HCT.

*Selectivity of the Method for Plasma Analysis.* For plasma analysis, selectivity was studied by analyzing 8 different plasma samples from the Blood Bank of Bezmialem Vakif University. The blank plasma samples did not yield any peak at the retention times of the analytes, when their chromatograms were compared with those obtained from spiked samples, indicating the absence of interferences and the high selectivity of the proposed method (Figure 4). 

## 5. Application to Patient Plasma Samples

**Figure 5:**
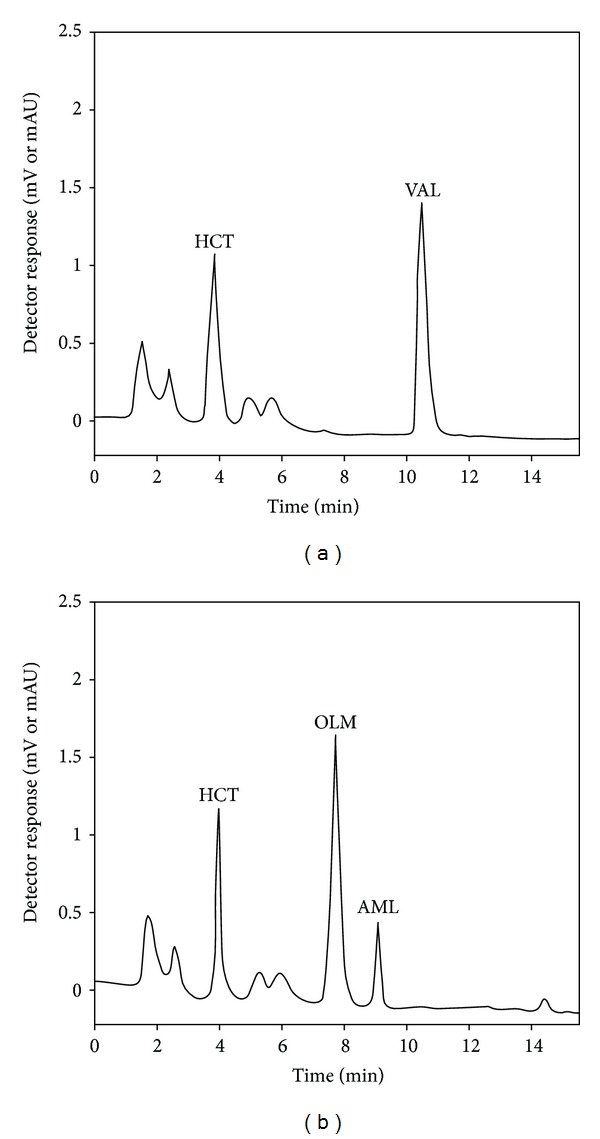
Chromatograms corresponding to plasma extracts of patients 1 (a) and 7 (b).

The developed method was applied to the plasma samples which were obtained from patients that used antihypertensive drugs, including the investigated compounds. The amounts of the drug substances found in the plasma of the patients are shown in Table 5. Besides, in Figure 5 it is possible to see some examples of chromatograms corresponding to plasma samples of patients under treatment with AML, OLM, VAL, and HCT in different combinations. So as to ascertain the purities of the peaks gained from the patients plasma samples, the active substances of the coadministered drugs added to the plasma samples of the patients and the same analytical procedures were repeated. No increase in the detection signals observed which displays that there is not any interference with the coadministered drug substances.

The proposed HPLC method is specific, accurate, and precise for the simultaneous determination of AML, OLM, VAL, and HCT. An important advantage of the method is the availability for the determination of the drug substances in pharmaceutical preparations and human plasma with a cost effective technique than other techniques including mass detection [19–27]. The chromatographic techniques combined with mass detection require high-cost equipment and therefore are not widely applied in routine laboratories. So it would be correct to say that it is suitable to utilize the presented method for the routine analysis of the drugs.

In addition, this method has other advantages over most of the previously published methods, such as its simplicity and less time-consuming procedure. Moreover, RSD and RME values of the method were very low, indicating high precision and accuracy.

 The pretreatment procedure is very simple, and it does not require any equipment like solid phase extraction cartridges or any more steps like double LLE. The main difference in the method compared to the previous ones is its ability to determine AML, OLM, VAL, and HCT simultaneously. 

## 6. Conclusion

In conclusion, the presented HPLC method is simple, selective, cost-effective, and reproducible and can be reliably used by almost every drug laboratory. The method enables simultaneous determination of AML, OLM, VAL, and HCT in pharmaceutical preparations and plasma. Due to the fact that these substances are mainly used as combinations for hypertension therapy, this new procedure is very important. In the process of developing the method, forced degradation and validation studies were carried out. Finally, the method was applied to the analysis for triple drug formulations, including *combinations* I and II and the quantification of the related substances in patient plasma samples.

## Figures and Tables

**Table 1:** Patients and administered drugs.

Patient number	Age	Gender	Drug^a^ and dose	Drug^b^ substance	Time period after last administration	Coadministered drug	Coadministered drug substance and dose
1	79	F	Co-Diovan 160 mg/12,5 mg	VAL, HCT	8	Aricept	5 mg dorepezil HCl
2	52	F	Norvasc 10 mg	AML	12	Mentopin Micardis	200 mg acetylcysteine 80 mg telmisartan
3	80	F	Norvasc 10 mg	AML	6	Glukofen	850 mg metformin HCl
4	63	M	Exforge 5/160 mg	AML, VAL	8	Ator	10 mg atorvastatin
5	71	F	Diovan 160 mg	VAL	8	Apranax	275 mg naproxen sodium
6	65	M	Hipersar Plus 20 mg/12.5 mg	OLM, HCT	7	Lipitor	20 mg atorvastatin
7	74	M	Sevikar HCT 40 mg/10 mg/25 mg	OLM, AML, HCT	8.5	Pletal	10 mg cilestazol
8	56	F	Cardopan Plus 80 mg/12.5 mg	VAL, HCT	24	Sirdalud	2 mg tizanidine

^a^Drugs that have been used for hypertension therapy.

^b^The active substances of the antihypertensive drugs.

**Table 2:** Parameters corresponding to linear regression obtained from the calibration curves for the plasma samples.

Drug substance	Slope ± SD	Intercept ± SD	Correlation coefficient	Linear range
OLM	31947	−5036.2	0.9995	0.4–25.6 *μ*g/mL
HCT	25436	−5846.7	0.9999	0.3–22 *μ*g/mL
VAL	36544	−1005.4	0.9998	0.3–15.5 *μ*g/mL
AML	34770	−10953	0.9998	0.1–18.5 *μ*g/mL

**Table 3:** Precision and accuracy of intraday and interday analyses at the low (1 *µ*g/mL for all substances), medium (10 *µ*g/mL for all substances), and high (15 *µ*g/mL for AML and VAL, 20 *µ*g/mL for OLM and HCT) concentration levels.

	Low (*μ*g/mL)		Medium (*μ*g/mL)		High (*μ*g/mL)	
	Intraday	Interday	Interday	Intraday	Interday	Intraday
RSD%						
AML	5.2	6.1	4.8	6.0	4.2	5.4
OLM	5.4	5.7	3.9	4.3	3.5	4.1
VAL	6.5	6.9	6.3	6.5	5.8	6.3
HCT	4.2	4.6	4.1	4.5	3.6	4.3
RME%						
AML	9.3	10.6	9.1	9.6	7.4	8.2
OLM	4.5	5.2	4.7	5.8	3.3	5.4
VAL	6.5	5.4	6.2	4.3	5.7	3.9
HCT	5.6	6.7	5.5	5.9	6.5	6.8

**Table 4:** Absolute and relative recoveries of plasma analysis.

	Concentration of AML (*μ*g/mL)			Concentration of OLM (*μ*g/mL)			Concentration of VAL (*μ*g/mL)			Concentration of HCT (*μ*g/mL)		
	0.5	10	18	0.5	15	25	0.5	10	15	0.8	10	20
Absolute recovery %	77.3	81.6	80.2	73.5	74.2	77.6	75.8	78	80.8	79.6	81.3	80
RSD%	4.5	3.7	3.5	2.6	2.5	1.8	4.2	3.9	2.5	2.7	1.7	1.6
Relative recovery %	95.5	97.6	97.9	92.6	95.8	96.7	100.3	99.8	98.7	92.7	100.3	100.1
RSD%	3.3	3.7	2.9	2.8	3.2	3.1	1.9	2.6	2.3	4.7	3.2	3.5

**Table 5:** Concentrations of the drug substances in the plasma samples obtained from patients (results given as mean values, *n* = 3).

Patient number	Time after administration	Concentration (ng/mL)			
		AML	OLM	VAL	HCT
1	8			1200	720
2	12	118.7			
3	6	152.3			
4	8	103.8		1000	
5	8			1242	
6	7		602.5		460
7	8.5	126	1023		920
8	24			352	412
